# Successful Implantation of Transvenous Pacing System via Persistent Left Superior Vena Cava and Coronary Sinus in Small Children

**Published:** 2011-02-07

**Authors:** Mohammad Dalili, Abolfath Alizadeh, Majid Haghjoo

**Affiliations:** Department of Pacemaker and Electrophysiology, Rajaie Cardiovascular Medical and Research Center, Tehran University of Medical Sciences, Tehran, Iran

**Keywords:** Pacing, Coronary sinus, Persistent left superior vena cava, Pediatrics

## Abstract

Transvenous pacemaker implantation tends to be difficult in the setting of a persistent left superior vena cava (SVC) and an absent or inaccessible right SVC. We report two small children in whom transvenous pacing leads were successfully inserted via a persistent left SVC. This technique was safe in our cases; however, favorable long-term result has yet to be demonstrated.

## Introduction

The route of choice for pacemaker implantation in small children remains controversial. The advent of small generators and low profile leads has made endocardial pacing more acceptable even for infants [1-3]. Nonetheless, there are some conditions where epicardial pacemaker implantation is not feasible and, as a result, transvenous pacing would be obligatory. The problem becomes more complicated when there is no normal venous pathway to the heart.

We herein present two small children in whom transvenous pacemaker implantation was successfully done in the setting of abnormal venous connections to the heart.

## Case 1

A 2-year-old boy weighing 10 kg developed atrial tachycardia early after the repair of the Tetralogy of Fallot. The atrial rate was fluctuating between 200 and 250, and the ventricular rate was about 90. Atrioventricular conduction could not be evaluated during the arrhythmia. The patient's general condition and his hemodynamic state were stable. Esophageal overdrive pacing and medical therapy were ineffective for the restoration of sinus rhythm. After two weeks, atrial and ventricular rates spontaneously decreased (converted to sinus rhythm) and atrioventricular block became more apparent. Electrophysiologic study confirmed sinus rhythm with complete heart block. Permanent pacing was indicated, but probable postoperative adhesions at that time made the epicardial lead insertion unfavorable.

**Figure 1:**
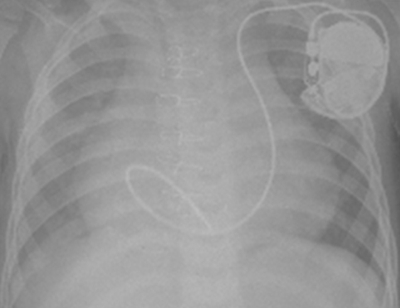
Chest X-ray shows transvenous active ventricular lead implantation via the persistent left superior vena cava and coronary sinus

The patient was transferred to the electrophysiology (EP) lab for pacemaker implantation. Under sterile condition and after appropriate sedation, the left subclavian vein (LSCV) was accessed. A guide wire was advanced into the vein; its course was through the left SVC into the coronary sinus and the right atrium. Venography from the peripheral right upper limb showed drainage of the right subclavian vein into the right atrium through the innominate vein, left SVC, and coronary sinus. The right superior caval vein was not opacified. An active fixation lead (Novus, 5076, 45 cm, Medtronic Inc, MN, USA) was introduced into the vein before it was looped in the right atrium and advanced into the right ventricle. The lead tip was fixed in the right ventricular apex (Figure 1). Early after implantation, a pacing threshold of 0.5 V @ 0.4 ms and the R-wave amplitude of 6 mV were obtained. The lead was connected to the generator (Zephyr, SR 5620, St. Jude Medical Inc., St. Paul, MN, USA), and the generator with attached lead was placed in the left subclavian pocket.

Pacemaker analysis three days, one month, three months, and six months after implantation indicated pacing thresholds of 0.5, 0.75, 0.5, and 0.5 V @ 0.4 ms, respectively. The R-wave amplitudes were about 5-7 mV in all the sessions. During this period, the patient remained asymptomatic and had no complication.

## Case 2

A 15-month-old girl weighing 9 kg was referred to our center with postoperative complete heart block. The patient developed heart block immediately after the total correction of Tetralogy of Fallot. Pacemaker implantation was postponed because of the patient's post-surgical sepsis. Ten day after cardiac surgery, the epicardial temporary pacemaker (TPM) became nonfunctional; therefore, a transvenous TPM was inserted via the right femoral vein. The patient's course became more complicated with transvenous TPM malfunction, deep venous thrombosis, cellulitis at right subclavian area, and left-sided pleural effusion. At that time, an epicardial permanent pacemaker was not a good choice because it was the time of maximal postoperative tissue adhesions.

**Figure 2:**
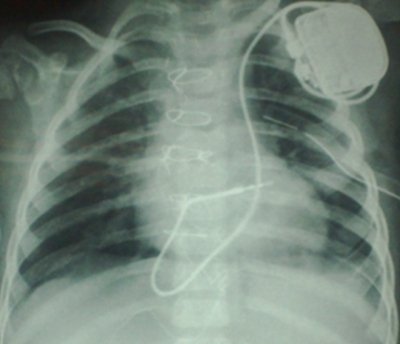
Chest X-ray shows endocardial pacemaker implantation via the persistent left superior vena cava and coronary sinus. Note that the active ventricular lead is fixed into the high interventricular septum

The patient was transferred to the EP lab in stable general condition. She was afebrile with a normal blood cell count, yet she had a left-sided chest tube. Under sterile condition and after adequate sedation, the LSCV was accessed. A guide wire was advanced into the vein but its course was abnormal. Venography from the LSCV access showed a persistent left SVC emptying into the right atrium via a dilated coronary sinus. Preoperative catheterization documented the presence of a right-sided SVC but the cellulitis at right subclavian area precluded right subclavian vein access. The innominate vein was not opacified during venography, and there was no pathway from the left subclavian vein to the RSVC. As was the case in our previous patient, an active fixation lead (Novus, 5076, 45 cm, Medtronic Inc., MN, USA) was advanced into the vein; it was looped in the right atrium and entered into the right ventricle. The active lead tip was fixed in the mideseptal area (Figure 2). The pacing threshold at implant was 0.6 V @ 0.3 ms; R-wave at that time was 9 mV. The lead was connected to the generator (Microny, 2525 T, St. Jude Medical, St. Paul, MN, USA) and it was located in the pocket in the left subclavian area via the routine method.  The patient was transferred to the intensive care unit in good general condition. The chest tube was removed two days later, and the patient was discharged from hospital three days after chest tube removal. Capture thresholds three days, one month, and three months after implantation were 0.25, 0.5, and 0.25 V @ 0.4 ms, respectively. The R-wave amplitude was about 8-10 mV in all the visits. The patient remained asymptomatic with no complications.

## Discussion

The present report shows successful implantation of the transvenous pacemaker implantation via persistent left SVC in two children weighing ≤10 Kg. In general, epicardial pacing systems are used in the infants and small children (< 10-15 kg) and those with complex congenital heart disease when anatomy precludes transvenous lead implantation. Epicardial lead implantation preserves the veins for future use and may be the only option for certain patients. The main problems are the fact that a sternotomy, thoracotomy, or subxiphoid approach is required, unipolar sensing/pacing systems are most commonly used, and some studies continue to suggest a higher rate of lead failure [4,5]. Furthermore, epicardial approach is not a good choice in the early postoperative period (with significant tissue adhesions) even in the small children. Efficacy and safety of transvenous pacemaker implantation in children has been demonstrated previously [6]. Transvenous permanent pacemaker implantation can be performed either from the right or from the left side, and because there are more right handed patients, the left side approach is more common.

Although the persistence of the left SVC results in no or minimal effect on the prognosis of the patient, it is of significance in situations such as transvenous lead insertion. Transvenous pacemaker implantation via a persistent left SVC and coronary sinus has been reported in adults with good results [7-9]. To the best of our knowledge, the present report is the first of its kind to show the feasibility of transvenous pacing lead insertion in the small children. Persistent left SVC is seen in about 0.3% of the general population [10]. In the presence of a congenital heart anomaly, the prevalence is much higher [10]. In approximately 60% of the cases, there is still a patent left innominate vein which connects the left SVC to the right SVC. In the other 40%, there is no venous connection between the left and right caval systems and the left SVC drains into right atrium via the coronary sinus. In rare cases, the right SVC is absent and the blood of the right system empties into the right atrium via the innominate vein, left SVC, and coronary sinus [10]. In cases with persistent left SVC and patent innominate vein, it is usually preferred to insert the lead via the innominate vein and right SVC. If the innominate pathway is not patent, it is usually preferred to insert the lead from the right subclavian area. In rare cases with an absent right SVC, however, the only way to the right atrium and right ventricle would be via the coronary sinus. In both cases presented herein, we were obliged to perform this technique. Negotiation of the lead tip from the right atrium into the right ventricle was very difficult. In both cases, we looped the lead in the right atrium as it tends to facilitate insertion and makes a reservation length for the child's growth. Both procedures lasted about 3 hours.

We did not implant dual-chamber pacemakers because of the higher risk of venous thrombosis [11,12] and no clear advantage of the dual-chamber pacemaker in terms of cardiac function [13] and pacemaker syndrome [14] in the small children.

Our experience supported the notion that the insertion of a transvenous pacing lead via a persistent left SVC and coronary sinus in small children is safe and effective. As in adults, the operator should be certain about the drainage of the left SVC into the right atrium before implantation. Long-term follow-up with a greater sample size is required to shed further light on the issue.
